# Hyperpolarized NMR Combined with Quantum Mechanical Simulations Reveal Atomistic Structures of Calcium Phosphate Prenucleation Clusters

**DOI:** 10.1021/acs.analchem.5c02945

**Published:** 2025-09-11

**Authors:** Christopher Pötzl, Ertan Turhan, Christel Gervais, Thierry Azaïs, Dennis Kurzbach

**Affiliations:** † Institute of Biological Chemistry, Faculty of Chemistry, 27258 University of Vienna, Währinger Str. 38, 1090 Vienna, Austria; ‡ Vienna Doctoral School in Chemistry (DoSChem), 27258 University of Vienna, Währinger Str. 42, 1090 Vienna, Austria; § CNRS, Laboratoire de Chimie de la Matière Condensée de Paris (LCMCP), Sorbonne Université, 75005 Paris, France

## Abstract

The discovery of solute precursors of crystalline materials, such as biominerals, recently challenged the classical nucleation theory (CNT). One emerging method for investigating these early-stage intermediates in solution is dissolution dynamic nuclear polarization (dDNP)-enhanced nuclear magnetic resonance (NMR) spectroscopy. Recent applications of dDNP to calcium carbonate (CaC) and calcium phosphate (CaP) mineralization have demonstrated the feasibility of identifying and tracing very early-stage prenucleation clusters (PNCs). However, the structural details remain difficult to resolve as dDNP is mainly limited to simple one-dimensional NMR detection. To overcome this bottleneck, we herein integrate hyperpolarized NMR of PNC with molecular dynamics simulations and quantum mechanical calculations to gain atomistic structural insights into CaP PNCs. By simulating the PNC structures, computing chemical shift parameters, and comparing these to hyperpolarized NMR “fingerprint” spectra, we demonstrate how to derive models of solution-state structural ensembles of PNC, even when very short-lived. With this approach, we find that the Ca/P_i_ ratio inside PNC tends to stay close to 1 independent of pH, while their sizes vary, leading to larger precursors under more basic conditions. At the same time, phosphate speciation within PNC was found to be independent of pH, as only monohydrogen phosphates participated in PNC formation. This latter feature also entailed a pH-independent local atomistic arrangement of phosphates coordinating a Ca­(II) center, leading to constant Ca^2+^–P_i_ distances of ∼3 and ∼3.6 Å. These ion-to-ion distances agree with those found inside solid CaP phases such as brushite, octacalcium phosphate, or hydroxyapatite—a feature hinting toward the templating function of PNCs. Thus, our method (i) extends the methodological scope of hyperpolarized NMR by complementing one-dimensional fingerprint spectra with full structural models and (ii) sheds light on key intermediates that have been experimentally underexplored.

## Graphical abstract

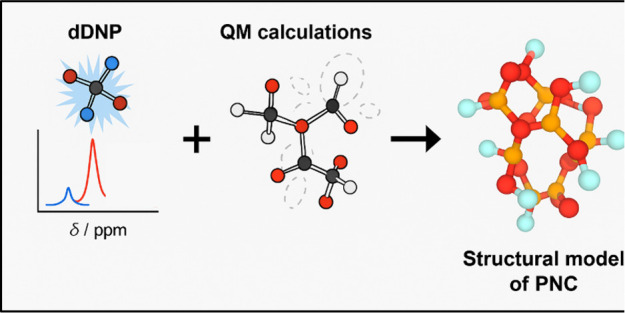

## Introduction

The recent discovery of early stage solution-state precursors of crystalline solids, including biominerals, has challenged the nucleation and growth paradigm of the classical crystallization theory (CNT). In particular, the study of prenucleation clusters (PNC) and the related potential nonclassical precipitation pathways (NCP) has become a topic of intense interest and discussion.^1–6^

The understanding of PNC is of high interest as it not only promises a fundamental update of our concept of material formation but also promises a route toward control over material formation mechanisms and, thus, rational materials design possibilities.^7,8^ Typically, these studies are undertaken under mild oversaturation conditions, which renders the precipitation kinetics slow and the study of PNC feasible, e.g., by Ca-potentiometry,^2,9–11^ in situ transmission electron microscopy,^12,13^ or conventional *in situ* spectroscopy including nuclear magnetic resonance (NMR).^14–17^ However, even when tailoring synthetic conditions to increase PNC longevity (typically from seconds to hours for CaP^6^) to ease their characterization, the composition, structure and internal dynamics of PNC remain largely undisclosed. This is not least due to a lack of methods that can comprehensively describe PNC given their complex structures and heterogeneous dynamics spanning various time and length scales.

When exiting the regime of mild oversaturation^6^ toward the large phase space at higher concentrations, the number of PNC reports becomes even more scarce.^18–20^ The rapid material formation kinetics and the resulting short PNC lifetimes render such studies experimentally very challenging.^21,22^

In this regard, one method that has recently received ample attention is dissolution dynamic nuclear polarization (dDNP)-boosted nuclear NMR spectroscopy.^18,20,22,23^ With dDNP, NMR signals can be enhanced often over 10,000-fold, allowing for rapid data acquisition within milliseconds to seconds after sample preparation. Thus, monitoring fast processes becomes possible (Figure 1) and it was shown that calcium carbonate (CaC) and phosphate (CaP) prenucleation could be tracked, and PNC identified on the time scale of only a few (≪10) seconds by means of such hyperpolarized NMR spectroscopy.^18,20,22,23,19,22^

**Figure 1:**
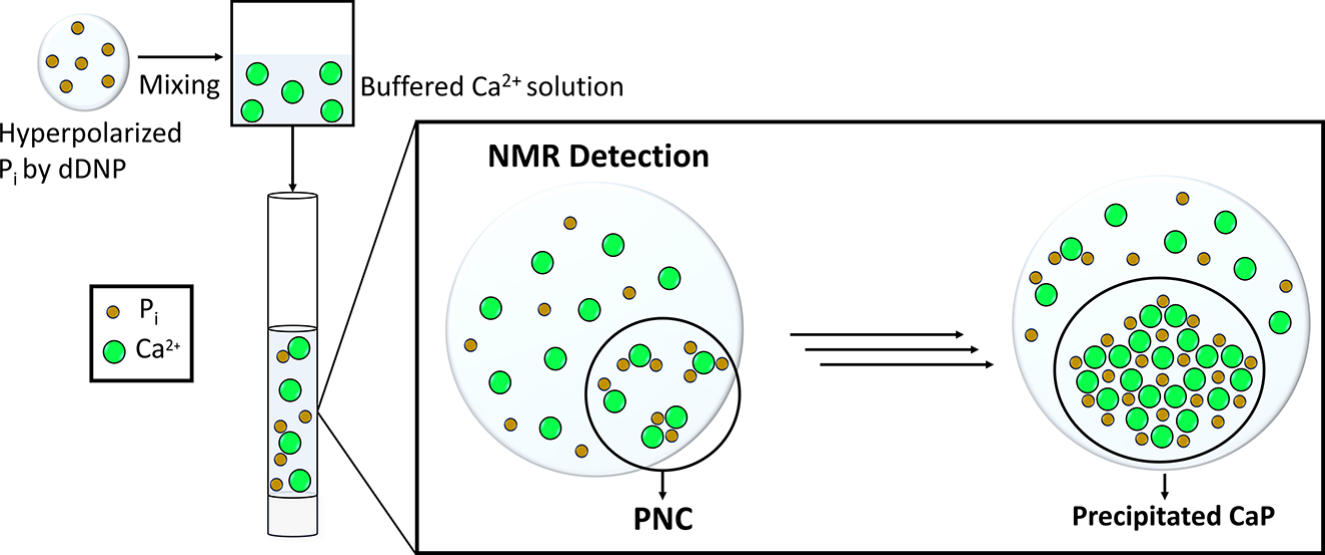
Overview of the dDNP NMR approach to monitor short-lived PNC via hyperpolarization of ^31^P nuclei in inorganic phosphate (P_i_). Mixing P_i_ with a Ca^2+^ solution initiates PNC formation, which is then immediately monitored by NMR on the single seconds time scale, to follow the formation of PNC preceding the precipitation of solid calcium phosphate.

However, rapid NMR read-out is only possible with the most straightforward experiments, i.e., mostly one-dimensional (1D) single-pulse spectra. Attempts to combine dDNP with 2D detection schemes, such as single-scan^24,25^ or the hyperpolarized water^26,27^ approaches, have not been reported yet in the context of NCP. Though powerful, their application to PNC is complicated by the rapid evolution of these species, often described as liquid-like ionic polymers.^1^

This constraint means that dDNP cannot simply derive the structure and the dynamics of PNC, such as NMR experiments like multidimensional NOESY or relaxation measurements. Only spectral 1D fingerprints of PNC are accessible, albeit with substantially enhanced signal intensity.

Herein, we demonstrate how to overcome this issue by combining the fingerprints of CaP PNC obtained by dDNP with molecular dynamics simulations and quantum mechanical chemical shift calculations. By simulating PNC, computing their spectroscopic properties, and comparing them against the hyperpolarized 1D spectra, precise structural models can be obtained and validated. Even very short-lived PNC over a wide range of solution conditions can be assessed in atomistic detail. Hence, we provide a tool to help rationalize the connection between PNC conformational ensembles in solution and the structure of the solid phase they precede.

With this integrative approach, we reveal important PNC features that have remained unobserved until now. In particular, we show that (i) the Ca^2+^/P_i_ (inorganic phosphate) ratio stays constant and close to 1, (ii) PNC sizes increase with rising solution pH, (iii) the local Ca^2+^-to-P_i_ coordination distances in PNC are found constant, i.e., pH-independent, and that (iv) these distances are in agreement with those found in the solid crystalline CaP phases such as such as brushite, octacalcium phosphate or hydroxyapatite. Hence, our data point toward a preformation of the local structures in solid CaP, i.e., the organization of phosphate ions around Ca­(II) centers, in PNC, already during the very early stages of material formation.

It should be noted that it is currently discussed whether the nanoscopic transient ionic species observed during the course of calcium carbonate and phosphate crystallization belong to the so-called nonclassical nucleation pathway (NCNP) or can be integrated in the CNT. For both perspectives arguments can be found in the current literature.^6,21,28–33^ For the presented study, it is, thus, important to consider that the proposed methodology supports neither theory but primarily reports a means to characterize early stage ion clusters in strongly oversaturated solutions preceding the formation of solid phosphate salts.

Our findings may thus open novel perspectives on CaP mineralization under strong oversaturation conditions - a part of the phase space that has been experimentally scarcely investigated - and may provide access to a previously undiscovered set of CaP properties.

## Materials and Methods

### Dissolution DNP

For DNP, all samples were prepared similarly: 0.5 M K_2_HPO_4_ were dissolved in a mixture of glycerol-d_8_ and H_2_O in a volumetric ratio of 15:85. TEMPOL was dissolved in the resulting solution at a concentration of 0.015 M. DNP was performed using the system described in reference^34^ at a temperature of 1.4 K and a magnetic field of 6.7 T for 2 h. The best build-up kinetics were observed using continuous microwave irradiation at 188.048 GHz. Dissolution with 5 mL pressurized D_2_O, transfer, injection, mixing, and degassing was fully automated within a home-built prototype similar to the one described in reference,^35^ leading to injections of 300 μL hyperpolarized sample in 1 s into a Shigemi NMR tube (without piston) waiting in the NMR spectrometer. To maintain the hyperpolarization during sample transfer, all fluid passages were sheltered with either Halbach magnets or pulsed solenoids. The ^31^P signals were detected simultaneously using θ = 8° flip angles for excitation with a repetition rate of 1 s^–1^ on a Bruker NEO 500 MHz spectrometer equipped with a BBFO Prodigy cryogenic probe. For the mixing experiments, Shigemi NMR tubes (Shigemi Co., Ltd.) were prefilled with 150 μL CaCl_2_ solutions (20 mM) buffered to pH 6, 7, or 8 with 0.1 M HEPES (pH 7 and 8) or MES (pH 6) in D_2_O. Upon injection of 300 μL the resulting sample volume was 450 μL. This procedure resulted in a final phosphate concentration of 12.8 mM and a final CaCl_2_ concentration of 6.7 mM, leading to [Ca^2+^]·[P_i_] = 85.76·mM^2^ and [Ca^2+^]/[P_i_] = 0.52.

For calcium pyruvate, a similar procedure was followed, yet the TEMPOL was replaced with 15 mM Ox064 radicals, and the signals were read out using ^13^C detection, exactly as described in reference.^35^ The final ratio was [Ca^2+^]/[Pyr] = 0.5, and the experiments were performed at pH 7.

In both cases, The D_2_O of the HEPES/MES buffer was used as lock solvent. The temperature variations upon mixing were below 1 °C. No pH variations were observed upon mixing given the high buffer strength.

All data were apodized using a 5 Hz exponential decay function, phased, and baseline corrected upon Fourier Transformation.

### MD Simulations

The calcium phosphate simulations were performed on a Workstation PC equipped with one Intel Core i9–12900KS processor (16*c*/24t), 64 GB DDR5–4800 MT/s RAM and one NVIDIA RTX 3090Ti GPU (Driver version: 515.65.01/CUDA version: 12.1). The Operating System was Rocky Linux 9 (Kernel version: 5.14) with the GCC/G++ compiler version of 11.2.) The GROMACS 2022.2 software package was used to set up and run the calcium phosphate simulation. The used force field was the all-atom additive CHARMM36 (July 2021 update) and was obtained from the MacKerell Web site.^36^ The TOPPAR files of HPO_4_^2–^ and H_2_PO_4_^–^ were obtained and converted to GROMACS file formats on the CHARMM-GUI Web site.^37^ The ions were placed in a cubic box with an edge length of 10 nm and solvated in water using the SPC/E water model.

All simulations contained 4 Ca^2+^ and 8 Cl^–^ ions, irrespective of the simulated pH value. The simulation for pH = 6 additionally contained 2 HPO_4_^2–^ ions, 6 H_2_PO_4_^–^ ions, 10 K^+^ ions, and 32717 H_2_O. The simulation for pH = 7 additionally contained 4 HPO_4_^2–^ ions, 4 H_2_PO_4_^–^ ions, 12 K^+^ ions, and 32719 H_2_O. The simulation for pH = 8 additionally contained 6 HPO_4_^2–^ ions, 2 H_2_PO_4_^–^ ions, 14 K^+^ ions, and 32724 H_2_O.

Energy minimization was performed using the steepest descent algorithm. The V-rescale thermostat was used for 200 ps of NVT equilibration. After NVT equilibration, the C-Rescale barostat was used for 200 ps of NPT equilibration. The duration of the production run under NPT conditions was 1000 ns, and the time step was 2 fs.

The calcium pyruvate simulations were performed on a Workstation PC equipped with one Intel Core i7–11700K Processor (8*c*/16t), 64 GB DDR4–3200 MT/s RAM and one NVIDIA GeForce RTX 3090. The GPU Driver version was 510.47.03 and the CUDA version was 11.5.

The ions were placed in a cubic box with an edge length of 5 nm and solvated in water using the SPC/E water model.

Energy minimization was performed using the steepest descent algorithm. The V-rescale thermostat was used for NVT equilibration for a duration of 300 ps. After NVT equilibration, NPT equilibration was performed using the C-Rescale barostat for 300 ps. The duration of the production run under NPT conditions was 400 ns and the time step was 0.2 fs.

### DFT Calculations

The DFT calculations were performed on two workstation PCs each equipped with one AMD Threadripper PRO 5995WX processor (64*c*/128t), one NVIDIA GeForce RTX 3090 Ti or NVIDIA GeForce RTX 4090, 128 GB DDR4–2666 MT/s ECC RAM. The NVIDIA HPC SDK 23.11 was used to take advantage of GPU support for structure relaxation, Born–Oppenheimer simulation and SCF calculations.

The GIPAW computations were performed on the same PCs used for the DFT, as well as on a node of the VSC-5 supercomputer equipped with two AMD EPYC 7713 processors (2x 64*c*/128t).

All DFT calculations were performed using Quantum Espresso 7.2 with a Bravais lattice index of 0 and a kinetic energy cutoff of 80 Ry. The David diagonalization was used with a convergence threshold of 10^–4^. The self-consistency convergence threshold was 10^–10^. In addition, charge density Broyden mixing with a mixing factor of 0.7 was used for self-consistency. PBE functionals were used for all atoms. The uniform k-point lattice was automatically generated.

The PBE functional was chosen due to its established accuracy in solid-state NMR shielding calculations via GIPAW, balancing computational cost and accuracy. While hybrid functionals such as B3LYP/PBE0 could improve absolute shift predictions, prior benchmarking studies^38^ suggest that PBE performs well for relative shift differences, which are central to our analysis. A plane-wave cutoff of 80 Ry was employed, ensuring a balance between computational feasibility and precision in shielding predictions. Furthermore, 35 snapshots extracted from MD were fully equilibrated over 200 ns before AIMD/DFT refinement using the same norm-conserving pseudopotentials as for GIPAW, ensuring robust sampling of relevant configurations.

The structure relaxation calculations were additionally performed with the BFGS quasi-Newton algorithm.

The Born–Oppenheimer calculations were performed with the Verlet algorithm, V-rescale thermostat at 298.15 K, and a second-order extrapolation. The time step was 4.8378.10^–17^ s, and a total of 100 steps were performed.

A key aspect is the averaging procedure to achieve the solution state structure. To compute ensemble-averaged isotropic magnetic shielding values (σ_iso_) representative of solution-state conditions, we employed a bootstrapping approach. Specifically, we extracted the last snapshot from each of 35 independent Born–Oppenheimer Molecular Dynamics (BOMD) trajectories. These BOMD simulations were initiated from structures obtained at evenly spaced intervals along the classical MD trajectory, following PNC formation.

### Ensemble Averaging

From the ensemble of 35 final BOMD snapshots, we conducted 1,000 bootstrapping iterations to estimate statistically robust ensemble-averaged isotropic shielding values, ⟨σ_iso_⟩. In each iteration, a resampled set of 35 values was drawn with replacement from the original σ_iso_ data, and the arithmetic mean of this sample was computed to yield a bootstrapped mean shielding value ⟨σ_iso_⟩_boot,i_. This process was repeated 1000 times to build a distribution of bootstrapped means. To ensure statistical convergence and eliminate early transients, we averaged only the final 15 iterations to obtain the final ensemble-average:

$$\langle \sigma_{\mathrm{iso}}\rangle =1/15\sum_{986}^{1000}\sigma_{\mathrm{iso},\mathrm{bootstrapping}\;\mathrm{mean}}$$ 1

The convergence of the running mean was verified by tracking the cumulative average over the 1,000 iterations. Additionally, the uncertainty in ⟨σ_iso_⟩ was estimated as the half-width of the 95% confidence interval (CI), divided by the *z*-score (*z* = 1.96):

$$\mathrm{Err}=\frac{1}{2z}({\mathrm{CI}}_{0.975}-{\mathrm{CI}}_{0.025})$$ 2

This bootstrapping scheme accounts for conformational diversity in the computed shielding values and provides a statistically grounded approximation of the solution-state NMR observable.

To compute the ^31^P shielding tensor of free phosphate in solution, the values for monohydrogen phosphate and dihydrogen phosphate were computed following the same strategy. Then the resulting ⟨σ_iso_⟩ values were weighted according to the Henderson–Hasselbalch relation to yield the average:

$${\left\langle \sigma_{\mathrm{iso}}\right\rangle }_{\mathrm{av}}=\chi {\left\langle \sigma_{\mathrm{iso}}\right\rangle }_{H2\mathrm{PO}4-}+\left(1-\chi \right){\left\langle \sigma_{\mathrm{iso}}\right\rangle }_{\mathrm{HPO}42-}$$ 3

$$\chi =c({{\mathrm{HPO}}_{4}}^{2-})/(c(H_{2}{{\mathrm{PO}}_{4}}^{-})+c({{\mathrm{HPO}}_{4}}^{2-}))$$ 4

Note that we define (as conventional) δ = σ_ref_ – σ_calc_, such that the chemical shift δ and the chemical shielding σ have opposite signs.

To achieve referencing of the computed chemical shift ⟨δ_iso,calc._⟩(^31^P), we measured the chemical shift of P_i_ at pH 4.5 using our buffer system, where almost pure H_2_PO_4_^–^ exists in solution.^39^ This led to δ­(^31^P) = 0.04 ppm corresponding to ⟨σ_iso_⟩(^31^P) = 281.1 ppm (cf. Figure S6).

### Simulation and GIPAW Calculation Setup of Calcium Pyruvate

*Ceteris paribus*, ions were placed in a cubic box with an edge length of 5 nm and solvated in water using the SPC/E water model. The simulation contained 20 pyruvate molecules, 10 Ca^2+^ ions, and 3960 water molecules.

## Results and Discussion

The approach reported herein is based on the acquisition of isotropic ^31^P chemical shifts of PNC by hyperpolarized NMR immediately (within 1 s) after sample preparation, i.e., after mixing of Ca^2+^ and P_i_ solutions. The experimental conditions were varied over a comprehensive pH range (6–8), while concentrations were chosen such that CaP precipitated very rapidly, preventing the use of alternative solution-state methods such as cryo-EM or SAXS. This requirement led to settings of strong oversaturation conditions often overlooked (here [Ca^2+^] × [P_i_] = 85.75 mM^2^, compared to mild oversaturation typically around 15–25 mM^2^).^10^

Importantly, the high ionic strengths allowed us to apply our numerical approach at concentrations that mirror the experiments exactly. Thus, we could directly correlate computationally derived structures with experimental data by simulating the ensemble-averaged chemical shift of the early stage CaP precursors in solution. To this end, we combined classical molecular dynamics (MD) simulations, ab initio molecular dynamics (AIMD) simulations, and Gauge-Including Projector Augmented Wave (GIPAW)^40^ NMR calculations of extensive conformational ensembles. The isotropic ^31^P chemical shielding of the phosphate ions resulting from the computation was then compared to experimental isotropic ^31^P chemical shifts obtained by hyperpolarized NMR through an unconventional bootstrapping resampling approach.

In the following, the experimental dDNP results will be described first, followed by the computational analysis.

### CaP PNC Detection by ^31^P Hyperpolarized NMR

Conceptually, all experiments were performed as described in reference.^23^ In brief, a freshly prepared solution^41^ of inorganic phosphate (P_i_) was flash-frozen and hyperpolarized ex-situ in a DNP apparatus (*B*_0,DNP_ = 6.7 T, *T*_DNP_ = 1.4 K), then dissolved and shuttled to an NMR spectrometer (*B*_0,NMR_ = 11.8 T, *T*_NMR_ = 298 K). There, the aqueous solution of hyperpolarized phosphate was collected in a prototype device (described in detail in ref 35 degassed, and mixed with a CaCl_2_ solution. The resulting solution was then injected into an NMR tube waiting in situ in the NMR spectrometer for detection (Figure 1). This procedure took only ∼1 s in total. In contrast to earlier applications,^18,23^ where the final sample was mixed directly in the NMR tube, this new procedure has the advantage that the detected solutions are perfectly homogeneous and free of gas inclusions. NMR detection thus also started directly 1 s after the first encounter of Ca^2+^ and P_i_ ions. A series of ^31^P 1D spectra was then recorded with a sampling rate of 1 s^–1^.

The P_i_ concentration upon detection was [P_i_] = 12.8 mM (as resulting from the dilution upon dissolution of the hyperpolarized sample; see the Materials and Methods), and the Ca^2+^ concentration was [Ca^2+^] = 6.7 mM (or naught for reference experiments). We probed three pH conditions with varying values of 6, 7, and 8 (both P_i_ and Ca^2+^ solutions were buffered by 50 mM TRIS or MES; see the Materials and Methods for details).

The signal enhancement was ε ∼ 1500 for the free P_i_ (Figure 2a). This corresponds to a 10^6^-fold reduction in measurement times, which allowed us to record the ^31^P spectra with a single scan and thus capture very short-lived PNC. This is important as, within seconds, the precursors are typically converted into solid CaP under the conditions probed herein, and all PNC resonances are lost.^18,23^

**Figure 2:**
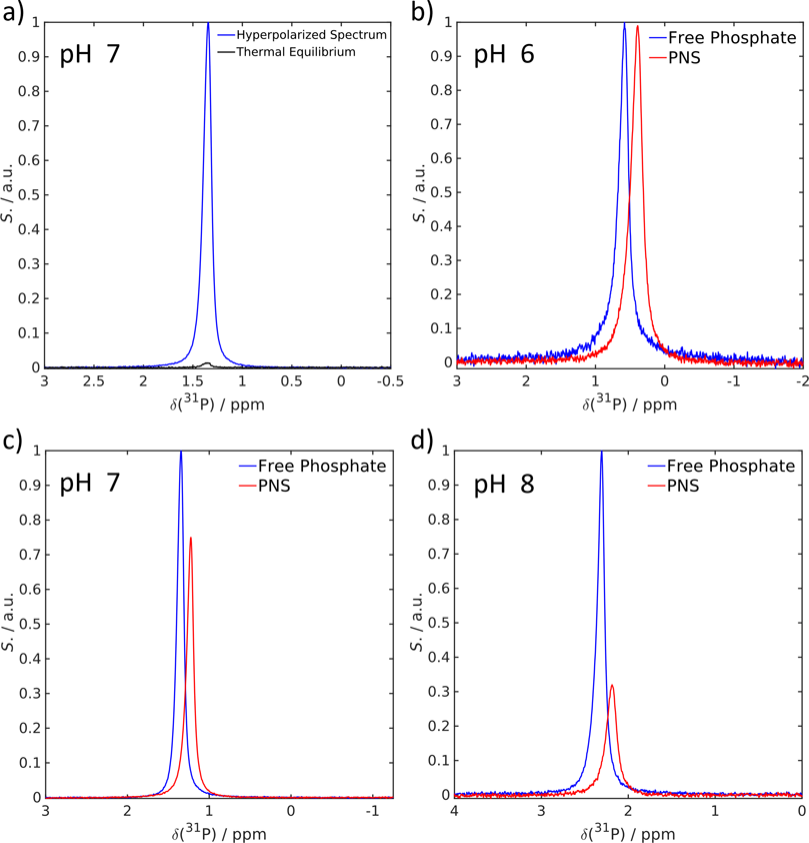
^31^P dDNP NMR spectra recorded 1 s after mixing Ca^2+^ and P_i_ solutions. (a) Comparison of hyperpolarized free phosphate (blue) and free phosphate at thermal equilibrium (20 scans averaged black) at pH = 7. (b–d) Comparison of hyperpolarized CaP PNC (red) and hyperpolarized free P_i_ (blue) at pH = 6 (b), 7 (c) and 8 (d).

For the same reason, no reliable spectrum under thermal equilibrium could be recorded in the presence of Ca^2+^, and no enhancement was determined.

Figure 2b,c shows the hyperpolarized ^31^P spectra immediately after mixing with a Ca^2+^ solution or in the absence of Ca^2+^. The encounter with Ca^2+^ ions immediately initiated the CaP mineralization process. Soluble, transient CaP precursors forming during the early stages of the material formation^18,21^ process were then witnessed through (i) changes in the ^31^P chemical shifts δ and (ii) in resonance line width Γ ; All values are reported in Table 1.

1. Regarding chemical shifts, we observed a consistent upfield shift Δδ, which decreased from ∼0.2 to ∼0.1 ppm when the pH was increased from 6 to 8. These chemical shift changes are also consistent with earlier observations under comparable conditions, which show the influence of P_i_ complexation with Ca^2+^ within PNC on the exchange-averaged ^31^P chemical shifts.^23^
2. Regarding resonance widths, we observed similar line broadening upon Ca^2+^ interaction in all three pH cases: an increase in line width (fwhm) of ΔΓ = 30 ppb. Such behavior was previously attributed to the slower tumbling of PNC-bound P_i_.^18,23,31^ At the same time, the signal-to-noise ratio (SNR) dropped compared to the reference obtained in a Ca^2+^-free buffer (Figure 2a).

**Table 1:** ^31^P Chemical Shifts and Resonance Line Broadening of Free (P_i_) and PNC-Bound Phosphate at the Probed pH

	δ_Pi_/ppm	δ_PNC_/ppm	Δδ/ppm	Γ_Pi_/ppb	Γ_PNC_/ppb	ΔΓ/ppb
pH = 6	0.58±0.01	0.39±0.01	–0.19±0.02	130	160	+30
pH = 7	1.34±0.01	1.22±0.01	–0.12±0.02	110	140	+30
pH = 8	2.32±0.01	2.20±0.01	–0.12±0.02	70	100	+30

Notably, the hyperpolarization decay curves also displayed faster decay due to precipitation during the detection period in the presence of Ca^2+^ relative to free P_i_ (Figure S1); again, in good agreement with previous studies.^18^

Finally, note that the ^31^P signals can result from P_i_ either in fast or slow exchange between free and PNC-bound states (on the NMR time scale), depending on concentrations, ionic strength, cosolutes, temperature, pH, and buffer.^14,18,23^ For the conditions probed herein, systems were consistently found to be in fast exchange. As a result, the differences between the free and PNC-bound phosphate signals in the hyperpolarized spectra appear relatively modest, though consistent and reproducible across pH conditions, and aligning with earlier reports.^18^ One might expect stronger effects based on prior studies at higher concentrations,^18^ yet the observed shifts remain significant and indicative of distinct coordination environments.

Taken together, the dDNP data clearly indicate the formation of CaP PNC. However, the key problem remains: their structures cannot be derived from the 1D fingerprint NMR spectra.

### Molecular Dynamics Simulations

To generate PNC models, we employed all-atom molecular dynamics (MD) simulations–a well-established tool in the investigation of NCP.^3,12,21,23,42,43^ Simulations were performed for all pH conditions as described in the Materials and Methods. Ca^2+^ and P_i_ ions were randomly distributed within a simulation box and energy-minimized in explicit water at the experimental pH, temperature, and concentration. Protonation states of P_i_ were set according to the experimental pH, maintaining the expected ratio of HPO_4_^2–^ to H_2_PO_4_^–^ (see Materials and Methods for details). It should be stressed that molecular dynamics (MD) simulations using our experimental concentrations ran at lower concentrations compared to most other reported computational approaches.^3,12,31,42,44,45^ This detail led to notable differences:

First, the time needed for PNC formation was relatively long on MD time scales. It took up to 800 ns for CaP PNC to complete their formation across all probed conditions. Figure 3a shows the RMSD of all Ca–P distances from the starting configuration. The formation of PNC is reflected by sudden changes of values (black arrows in Figure 3a indicate the completion of the PNC formation events). The PNC manifested as spatially dense ion clusters (Figure 3b). The formation was further evidenced by the emergence of stable ion–ion contacts with defined distances at ∼3 and ∼3.6 Å that persisted until the end of the simulation (see Supporting Information, Figures S2–S4). The rapid formation of these species, driven by long-range electrostatic attraction, provides a mechanistic rationale for the dDNP experiments, which detected PNC under all tested conditions within the first scan.

**Figure 3:**
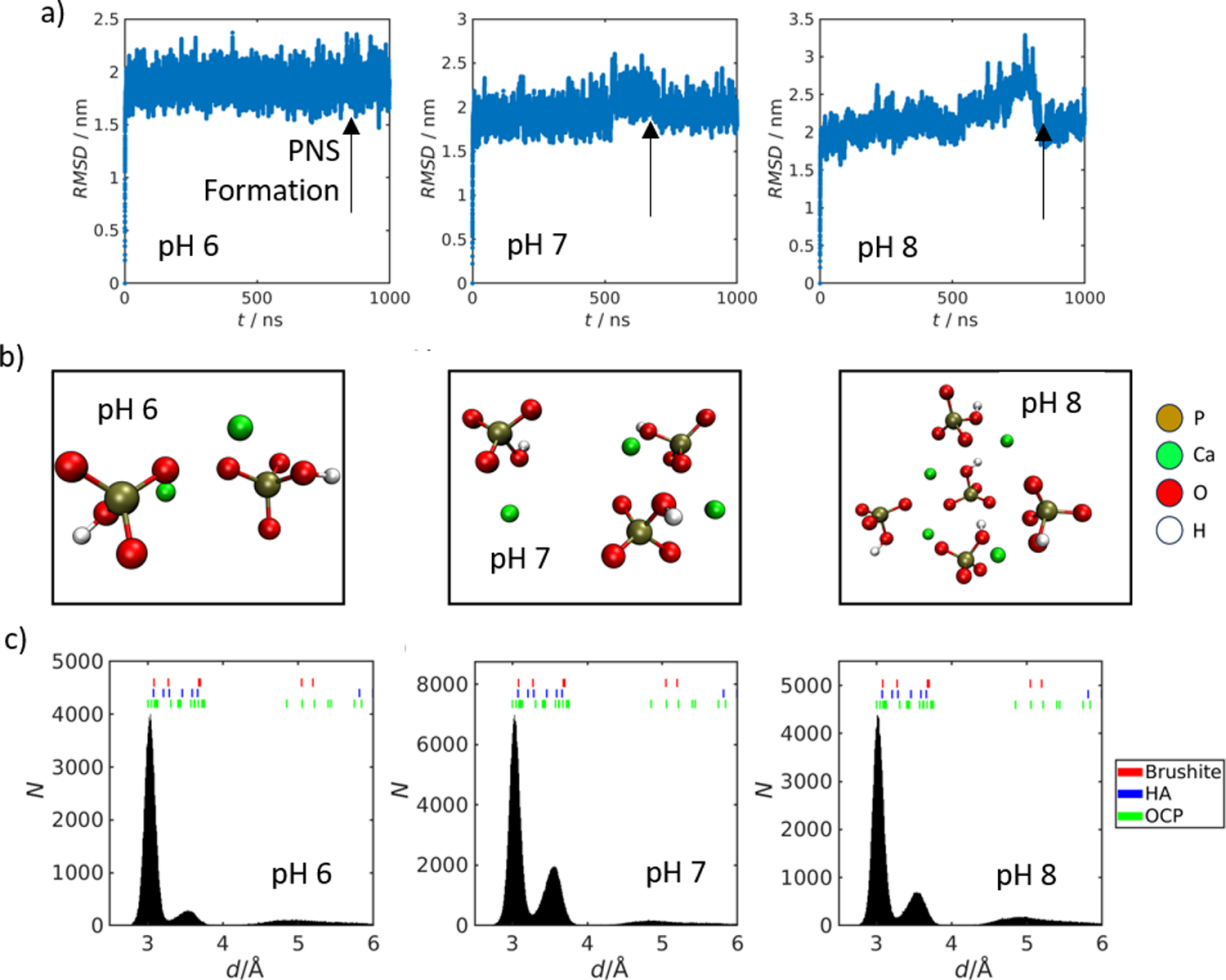
(a) RMSD (all Ca–P distances relative to the starting configuration) trajectories for pH 6, 7, and 8 with the moment of completion of PNC formation indicated. (b) Representative PNC structure (snapshots) formed at the different pH values. (c) Distance histograms for all three probed pH conditions. The distances found in OCP, HA and brushite crystals are indicated as colored dashes.

The second unexpected observation was the constitution of the PNC, composed of Ca^2+^:P_i_ ratios of 2:2, 3:3, and 4:5 (Figure 3b). These neutral or almost neutral PNC contrast with the often-reported calcium triphosphate (1:3) charged cluster.^19–22^ The lower concentrations used for the simulation likely led to changes in PNC conformation, as it is well established that PNC structures and conformational spaces depend heavily on solution conditions.^19–22^

The third striking observation was the binding of 7, 10, and 14 water molecules, respectively (hydration water within a 0.27 nm radius around each Ca^2+^ ion)^46^ leading to a relatively constant H_2_O/Ca^2+^ molar ratio in PNC of 3.5, 3.33 and 3.5, respectively, confirming that the hydration degree is a key structural characteristic of PNC.

Finally, we observed that exclusively HPO_4_^2–^ species contributed to the formation of PNC. H_2_PO_4_^–^ species remained in solution in all simulated cases. The stronger electrostatic potential of the divalent species overruled the influence of the shifting pH-dependent HPO_4_^2–^/H_2_PO_4_^–^ ratio. This is an important observation confirming that solid crystalline phases embedding H_2_PO_4_^–^ species, such as monocalcium phosphate monohydrate Ca­(H_2_PO_4_)_2_·H_2_O, are not favored at the investigated pH, independent of the presence of H_2_PO_4_^–^ species in solution.^47^ Hence, it appears that phosphate speciation in CaP phases can be reflected in the constitution of the PNC.

To comprehensively reflect the complex conformational space of the PNC found in the MD simulations over the range of probed pH conditions, we computed the distance distributions between all ^31^P nuclei within the HPO_4_^2–^ species forming PNC (representing the center of gravity of the phosphate ions), and all Ca^2+^ ions for each trajectory. The resulting distributions are shown in Figure 3c. Two key observations merit highlighting:

1. The distributions consistently exhibit maxima at similar distances (∼3 and ∼3.6 Å), with differences observed only in relative populations. In other words, the local arrangement of the phosphate ions around the Ca^2+^ ions in terms of distances remains unchanged across the tested pH conditions. These distances correspond to the van der Waals-type ion-to-ion contacts (3 Å) or stem from conserved geometry relative to the second next P_i_ unit (3.6 Å).
2. The Ca^2+^ distances obtained from the MD simulations align qualitatively with those found in solid-phase CaP structures formed under our experimental conditions,^18,23,48^ including brushite, octacalcium phosphate (OCP), and hydroxyapatite (HA) (colored lines in Figure 3c). These findings are in agreement with previous work by Mancardi et al.,^31^ supporting the idea that phosphate coordination in solid CaP phases is, at least partially, preconfigured at the early stage PNC levels.

Thus, the MD simulations alone suggest interesting features, namely the preselection of P_i_ species and preformation of ion-to-ion distances. Yet, these data need to be experimentally verified to prove a reliable source of information.

### Correlating Hyperpolarized NMR and MD Simulations

By quantitatively matching the MD simulation results and the hyperpolarized NMR fingerprints, a 2-fold advantage can be gained. First, the NMR data can be complemented with structural information, and second, the computational results can be experimentally validated.

To achieve this synergy, we combined the MD results with Born–Oppenheimer ab initio MD (BOMD) simulations to generate computational structural ensembles with energy-minimized bond lengths. These ensembles were then used as input for density functional theory (DFT) calculations to

1. compute the isotropic ^31^P magnetic shielding values σ_iso_ of free P_i_ (HPO_4_^2–^, H_2_PO_4_^–^ in the absence of any Ca^2+^) and of CaP PNC formed at the three different probed pH conditions.
2. compare the computed shielding values with dDNP-derived chemical shifts before and upon PNC formation (Tables 1 and 2).

**Table 2:** Isotropic ^31^P Shielding and Chemical Shift Values of Weighted Average between H_2_PO_4_^–^ and HPO_4_^–2^ and within PNC at pH = 6, 7, and 8

	⟨σ_iso_⟩(P_i_)/ppm (weighted)	⟨σ_iso_⟩(P_PNC_)/ppm	⟨δ_iso_⟩(P_i_)/ppm (weighted)	⟨δ_iso_⟩(P_PNC_)/ppm
pH = 6	280.9 ± 0.5	278.9 ± 0.4	0.35 ± 0.5	2.38 ± 0.4
pH = 7	279.3 ± 0.4	278.7 ± 0.2	1.98 ± 0.4	2.55 ± 0.2
pH = 8	278.7 ± 0.3	278.4 ± 0.3	2.63 ± 0.3	2.84 ± 0.3

However, the challenge lies in the fact that solution-state NMR, as used for detection in dDNP experiments, reports on samples in motion, and thus, only on dynamically ensemble-averaged chemical shift values. Hence, ensemble averaging should also be considered for chemical shift calculations before comparing computed chemical shifts with experimental data. Earlier approaches to computing liquid-state chemical shifts via ensemble averaging have been successfully demonstrated.^49–53^ The key distinction of our method is the implementation of a bootstrapping-based analysis to enforce statistical convergence, even in cases of strongly fluctuating chemical shielding tensors.

To this end, we extracted 35 evenly spaced snapshots from the classical MD simulations after the formation of stable PNC and isolated the PNC clusters. Each cluster was then solvated in explicit water within a cubic simulation box (edge length: 1.1 nm) and equilibrated for 5 ns under NVT conditions at 298 K. The equilibrated systems were subsequently optimized using quantum mechanical relaxation (see Materials and Methods), employing PBE functionals within Quantum Espresso 7.2. BOMD simulations were then performed for 100 steps using a step length of 0.5 fs.

The structures resulting from the BOMD runs were then used to compute magnetic shielding tensors σ using the GIPAW module in Quantum Espresso 7.2.

To obtain isotropic shielding values, we computed the σ tensor for the last trajectory step of each DFT run originating from the 35 classical MD structures. We then took the isotropic average σ_iso_ over the σ tensors’ diagonals.

To approximate solution-state NMR conditions, we performed 1,000 bootstrapping iterations by resampling (with replacement) these σ_iso_ values, computing the mean for each, and averaging the final 15 bootstrapped values to yield statistically robust ensemble-averaged shielding values (details in the Materials and Methods and Figure S5). We averaged over the last 15 iterations to obtain the ensemble-average value ⟨σ_iso_⟩. Figure 4a shows the workflow and the resulting ⟨σ_iso_⟩ values are reported as histograms in Figure 4b for each pH condition. The final chemical shielding values were then considered as the means of the histograms shown. These values are also reported in Table 2. Our approach ensured that sufficient equilibrated snapshots were fed into our GIPAW calculations to approximate solution-state NMR conditions. Note that similar approaches have already been successful in other contexts^54^ and are herein expanded to ^31^P NMR of PNC.

**Figure 4:**
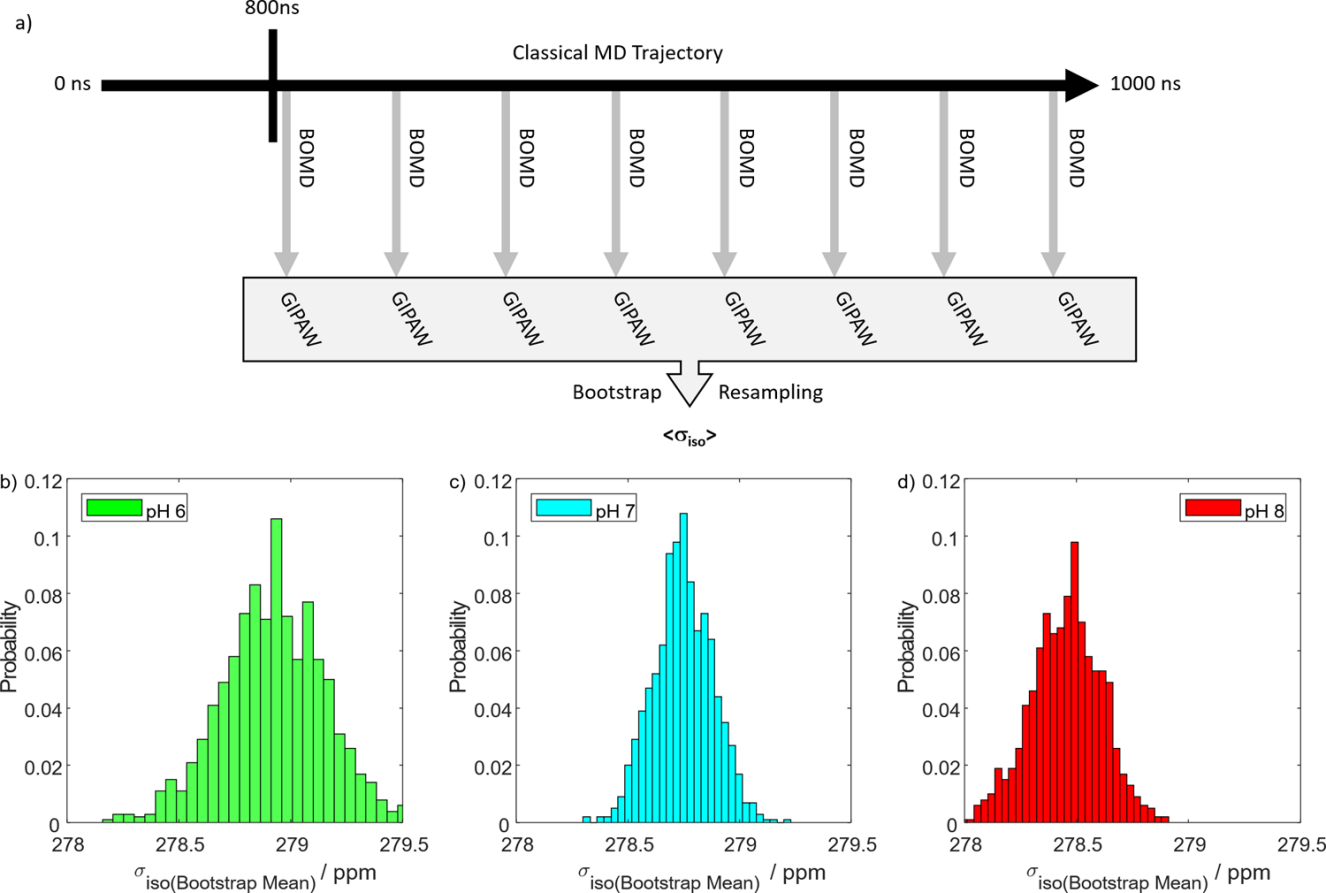
(a) Scheme of the workflow used to compute ensemble-averaged chemical shielding values. (b–d) Comparison of isotropic magnetic shielding values for all probed pH values. The means of these histograms correspond to the used ⟨σ_iso_⟩ (see Supporting Information Figure S5 for a convergence analysis).

To fully match the computational data with the experimental chemical shifts, we also computed ⟨σ_iso_⟩ values for free HPO_4_^2–^, H_2_PO_4_^–^ in a similar fashion, i.e., combining classical MD and BOMD (see Supporting Information Figure S6). To obtain the shielding at the experimental pH values, we then considered population-weighted contributions of the two values for HPO_4_^2–^ and H_2_PO_4_^–^ based on the Henderson–Hasselbalch relation to compute a population-weighted ⟨σ_iso_⟩ (see the Materials and Methods for details). This approach ensured that at each probed pH, the relative contributions of the phosphate species were appropriately accounted for, reflecting their equilibrium distribution in solution. Finally, we converted the chemical shielding values into chemical shifts using δ­(^31^P) of H_2_PO_4_^–^ at pH 4.5 for referencing (all details in the Materials and Methods).

With the chemical shift values for free P_i_ and PNC-bound P_PNC_ in hand, we could move on to experimental cross-validation. All results are comprehensively shown in Figure 5. Figure 5a,b show the pH-dependence of experimental chemical shifts and the computed values. For the latter case, errors have been determined via the width of the distributions shown in Figure 4. Figure 5c,d compare the computed and experimental values for P_i_ and PNC-bound P_PNC_ with respect to pH, while Figure 5e,f directly plot the experimental shifts vs computed isotropic, averaged chemical shift values.

**Figure 5:**
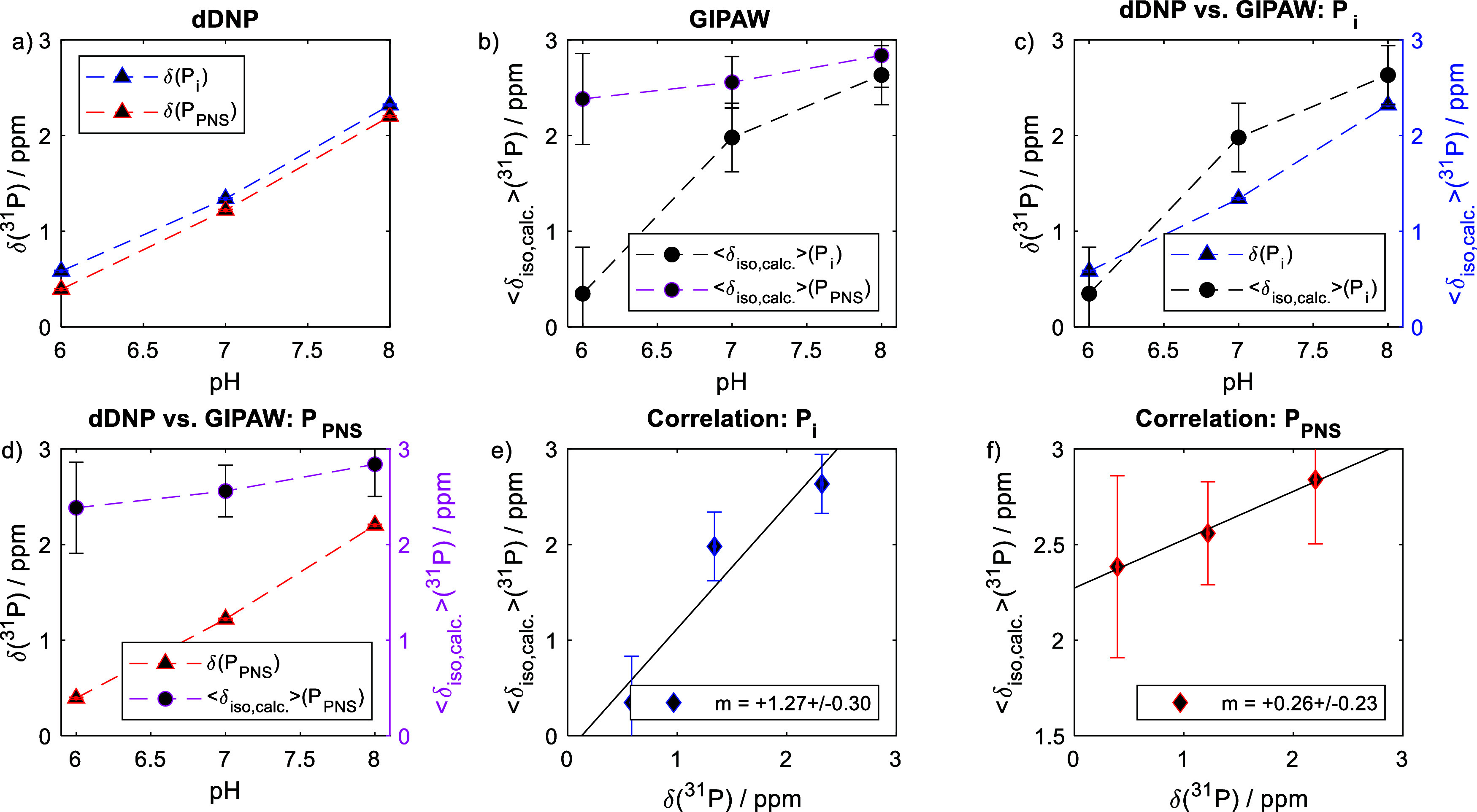
(a) pH-dependence of the experimental chemical shifts of phosphates in solution and within PNC (P_i_ and P_PNC_). (b) pH-dependence of the computed, averaged chemical shift values. (c) Comparison of the computed and measured values for free P_i_. (d) Comparison of the computed and measured values for P_PNC_. (e) Correlation plot of measured and computed values for free P_i_. (f) Correlation plot of measured and computed values for P_PNC_. The black solid line corresponds to a linear fit. The slope *m* is indicated in the figures.

As evident from Figure 5a–d, the computed ⟨δ_iso_⟩ values track the experimental δ­(^31^P) trends, with both data sets exhibiting a monotonic downfield shift with rising pH both for phosphate in solution (P_i_) (Figure 5c) and within PNC (P_PNC_) (Figure 5d). This agreement supports the robustness of our computational approach in capturing the underlying chemical environments driving the NMR chemical shift evolution upon pH variation and complexation to Ca^2+^.

Moreover, Figure 5c further evidence that our computed values for P_i_ almost quantitatively agree with the experiment. In contrast, even if the overall trend is correctly computed, a difference between simulation and experiment was observed for P_PNC_ (Figure 5d). The computed values do not show a similarly strong pH dependence as experimental ones. Indeed, a variation of 1.81 from pH 6 to 8 is observed experimentally, whereas a variation of only 0.50 ppm is observed computationally. This likely originates from the fact that the computed chemical shift values reflect only the PNC-bound phosphate population, whereas the experimental δ­(^31^P) values are population-averaged under fast exchange conditions (vide supra), including contributions from both free and bound species. A difference between computation and experiment must, thus, be considered. This feature is also reflected in the correlation plots shown in Figure 5e,f, where the slope for free P_i_ approaches unity, while that of P_PNC_ remains smaller than unity.

As such, while the computationally derived chemical shifts provide a meaningful structural interpretation of the experimental data, their exact numerical agreement with measured values should be considered in a qualitative rather than strictly quantitative manner.

While the experimentally observed chemical shifts likely reflect population-weighted averages under fast exchange conditions, we refrained from estimating relative populations of free and bound phosphate species, as such a calculation would require uncertain assumptions regarding local pH, protonation states, and species distributions. Given the computational uncertainties and limited impact on the overall conclusions, we chose to report directly computed values for the individual species.

Nonetheless, the chemical shift obtained from the computed structures correlated with the experimental values within the precision of the presented approach. This correlation indicates that the simulated structures can well satisfy the experimental spectral parameters. Hence, the hyperpolarized fingerprints can similarly be underpinned by a cross-validated structural model.

Note that, in contrast to GIPAW, DFT calculations using nonperiodic boundary conditions have recently been reported to perform well in solution-state chemical shift calculations.^55–57^ Thus, we tested these methods for the present case of δ­(^31^P) in P_i_, but the results did not correlate well with our experimental values. Such approaches, e.g., coupled-cluster methods, employ nonatomistic solvent models and hydrogen bonds, as well as solvent coordination, which decisively impact chemical shifts for P_i_, which are not fully considered. Hence, while methods such as coupled-cluster simulations might be superior in aprotic systems, we found explicit solvents to perform better for our work case.

Expanding our approach to ^13^C-detected NMR and the case of Ca^2+^ complexation by pyruvate lead to quantitative agreement between experiment and simulation, further confirmed the broader applicability of our method (see the Supporting Information).

## Conclusions

Our dDNP experiments reveal that under strong oversaturation conditions, early stage CaP precursors form within <1 s of mixing phosphate and Ca^2+^ ions, across a wide pH range. These transient species can be reliably detected by hyperpolarized NMR. By comparing experimental data with MD simulations and quantum mechanical chemical shift calculations, we demonstrate that it is possible to derive structurally validated atomistic models of PNC, even for such short-lived species.

Our earlier work demonstrated that CaP PNC exhibit heterogeneous structural dynamics on different time and length scales in the sense that individual CaP nanoclusters tend to aggregate in supramolecular assemblies of hundreds of nm in solution with time.^48^ In these systems, NMR is sensitive primarily to the smallest building blocks of PNC. Since NMR-accessible species are typically limited to low molecular weights, the PNC observed in dDNP experiments likely represent the smallest functional unit of larger prenucleation structures, potentially in exchange between PNC-bound and unbound states.^14^ A similar pattern emerges in our MD simulations, where the structures formed on short nanosecond time scales correspond to the smallest CaP clusters that appear earliest in the nucleation process. The agreement between hyperpolarized NMR data and computed chemical shifts suggests that this sensitivity to short-length scales is a key factor in the observed correlation.

A key insight from our methodological approach is the experimental validation that the local atomic coordination within these smallest PNC units is pH-independent in terms of phosphate speciation and in terms of ion-to-ion distances. Only the number of coordinated ions increased with solution conditions becoming more basic, with PNC composed of 2:2, 3:3, and 4:5 ratios of Ca^**2**+^ to P_i_. Moreover, the number of coordinated water molecules to Ca^**2**+^ stays constant within our conditions, highlighting the key role of the hydration level of PNC. Indeed, controlled dehydration is proposed to drive PNC aggregation and evolution to denser phases.^58^

Importantly, the Ca^2+^-to-P_i_ distances closely resemble those found in solid CaP for all probed conditions. Together with the fact that only monohydrogenated phosphate species contribute to the formation of PNC, this finding suggests that aspects of the final solid-state structure are already preconfigured in the earliest solution-state precursors, reinforcing the concept of nonclassical nucleation pathways.^3,31^

The ability to map PNC structure–function relationships opens new possibilities for rational material design and biomineralization studies. Future work will focus on expanding this methodology to other mineralization pathways and biologically relevant environments.

## Supplementary Material



## Notes

Supplementary dDNP and MD data and plots; and NMR and computational data on calcium pyruvate (PDF)
