# Supplementary material for: Hyperpolarized NMR Combined with Quantum Mechanical Simulations Reveal Atomistic Structures of Calcium Phosphate Prenucleation Clusters
Source: Anal Chem. 2025 Sep 11;97(37):20191–200. doi: 10.1021/acs.analchem.5c02945 (PMC12461679; doi:10.1021/acs.analchem.5c02945)
Supplement: Supplementary file 1 [file ac5c02945_si_001.pdf]

Hyperpolarized NMR Combined with Quantum Mechanical Simulations Reveal Atomistic Structures of Calcium Phosphate Pre-nucleation Clusters

Christopher Pötzl[1,2,+], Ertan Turhan[1,+], Christel Gervais[3], Thierry Azaïs[3], Dennis Kurzbach[1]*

[1]Institute of Biological Chemistry, Faculty of Chemistry, University of Vienna, Währinger Str. 38, 1090 Vienna, Austria
[2]University of Vienna, Vienna Doctoral School in Chemistry (DoSChem), Währinger Str. 42, 1090 Vienna, Austria
[3]Sorbonne Université, CNRS, Laboratoire de Chimie de la Matière Condensée de Paris (LCMCP), 75005 Paris, France
[+] equal contributions
** dennis.kurzbach@univie.ac.at*

Supporting Information

Table of Contents

Figure S 1. Signal decay curves 2
Figure S 2. Ca^{2+}-^{31}P distances....2
Figure S 3. Ca^{2+}-^{31}P distances....3
Figure S 4. Ca^{2+}-^{31}P distance....3
Figure S 5. Cumulative mean convergence of $\langle\sigma_{iso}\rangle$ 4
Figure S 6. Isotropic shielding distribution histogram 4
Figure S 7. Distribution histograms of the calculated magnetic shielding 5
Figure S 8. Cumulative mean convergence plots 5
Figure S 9. ^{13}C NMR spectrum of sodium pyruvate in D_2O....5
Figure S 10. Correlation plot 6
Figure S 11. Results on Ca^{2+}-pyruvate 7

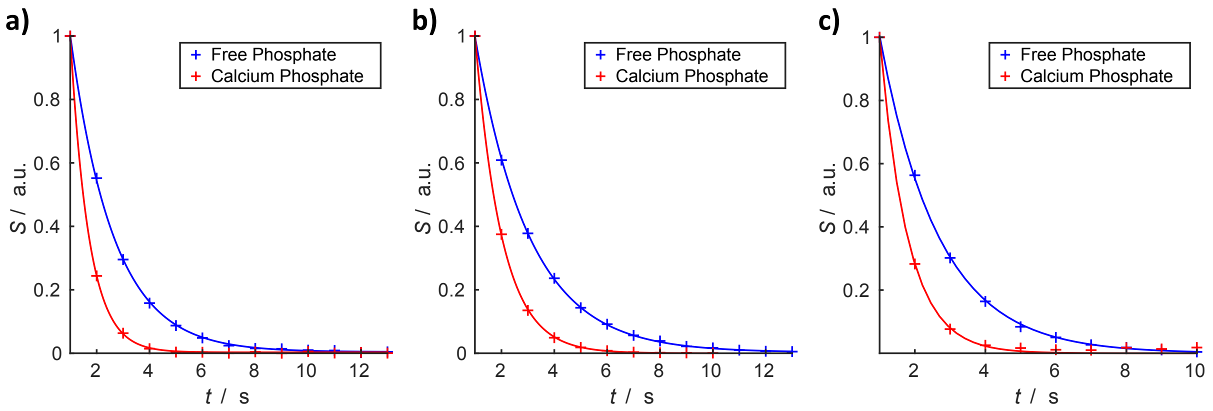

Figure S 1. Signal decay curves of free (blue) and PNS-bound phosphate (red) at a) pH = 6, b) pH = 7, and
c) pH = 8.

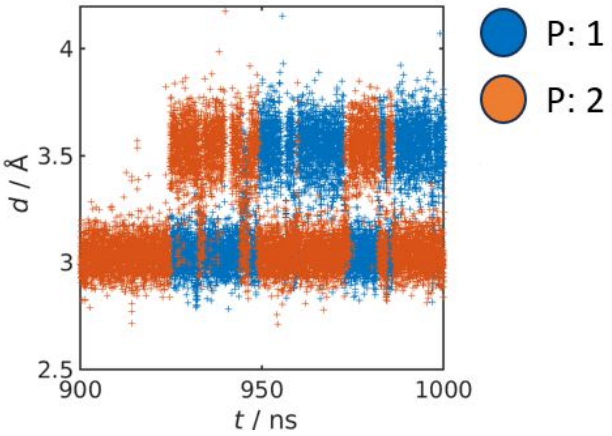

Figure S 2. Ca^{2+}-^{31}P distances as a function of simulation time in the MD simulation at pH = 6 for selected phosphate ions (see color code). The emergence of a constant distance between phosphates and a Ca^{2+} ion indicates the formation of PNS. Note that ions can switch places.

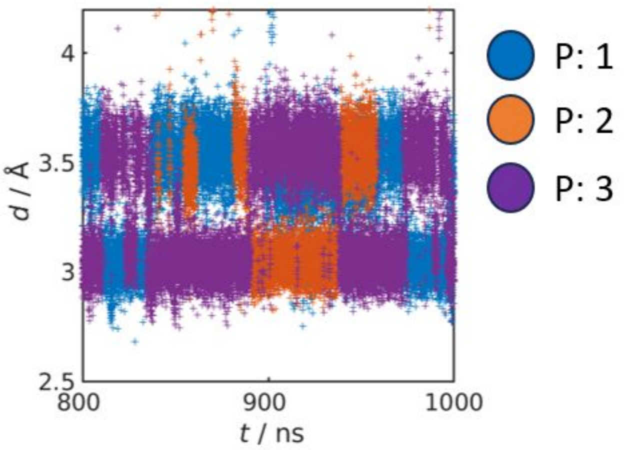

Figure S 3. Ca^{2+}-^{31}P distances as a function of simulation time in the MD simulation at pH = 7 for selected phosphate ions (see color code). The emergence of a constant distance between phosphates and a Ca^{2+} ion indicates the formation of PNS.

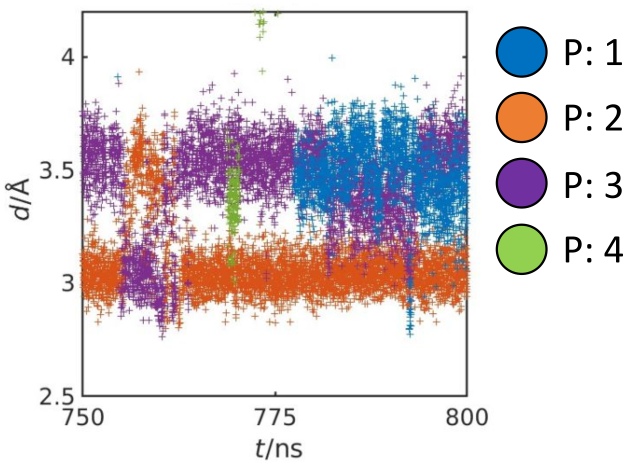

Figure S 4. Ca^{2+}-^{31}P distances as a function of simulation time in the MD simulation at pH = 8 for selected phosphate ions (see color code). The emergence of a constant distance between phosphates and a Ca^{2+} ion indicates the formation of PNS.

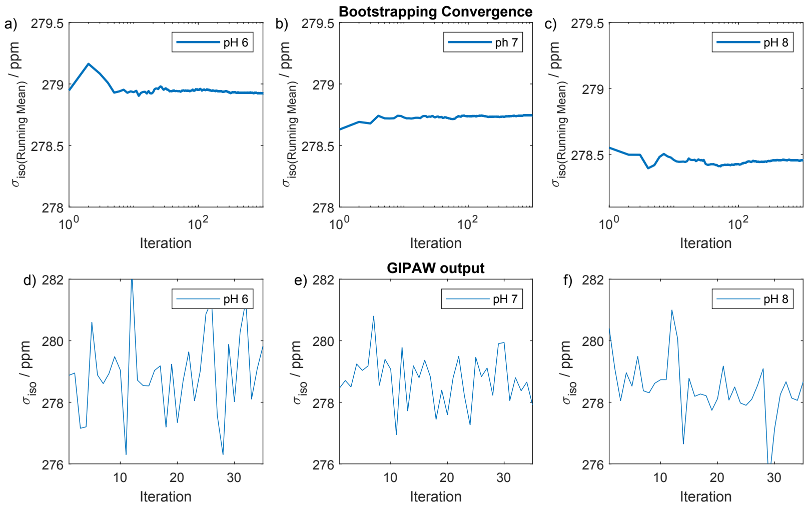

Figure S 5. Cumulative mean convergence of ⟨σ_{iso}⟩ for GIPAW calculations for a) pH = 6, b) pH = 7 and c) pH = 8 (*progressive average with increasing number of structures) and isotropic s*hielding σ obtained from the GIPAW calculations for a d) pH = 6, e) pH = 7 and f) pH = 8

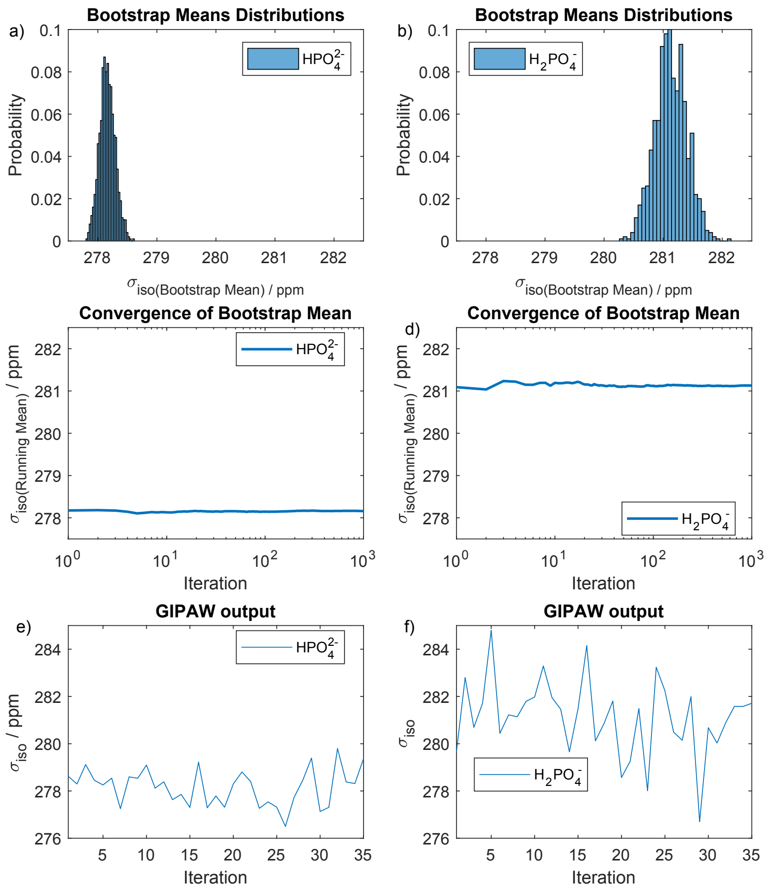

Figure S 6. Isotropic shielding distribution histogram of a) K_2HPO_4 and b) KH_2PO_4 GIPAW calculations. Cumulative mean convergence plots of ⟨σ_{iso}⟩ for c) K_2HPO_4 and d) KH_2PO_4. Plot of the magnetic shielding σ obtained from the GIPAW calculations for e) K_2HPO_4, f) KH_2PO_4,

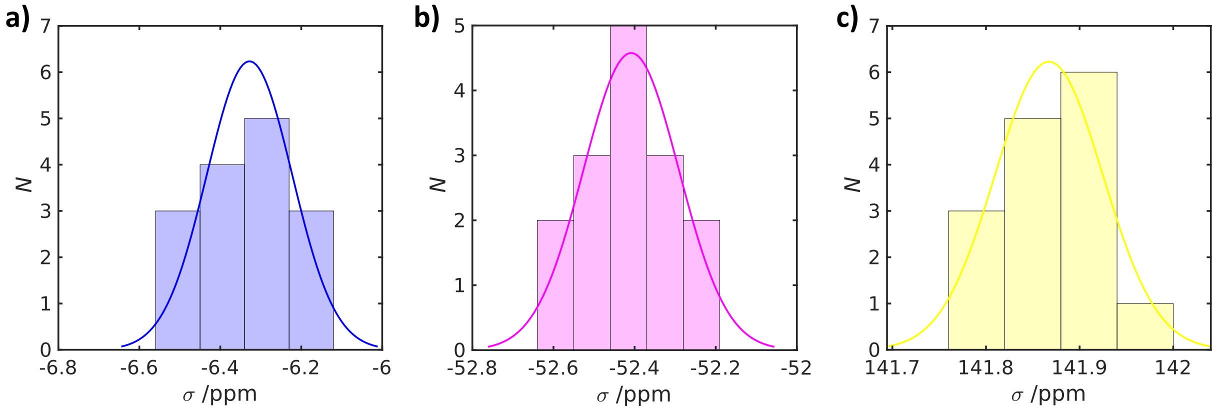

Figure S 7. Distribution histograms of the calculated magnetic shielding σ of a) C-1, b) C-2, c) C-3 for potassium pyruvate.

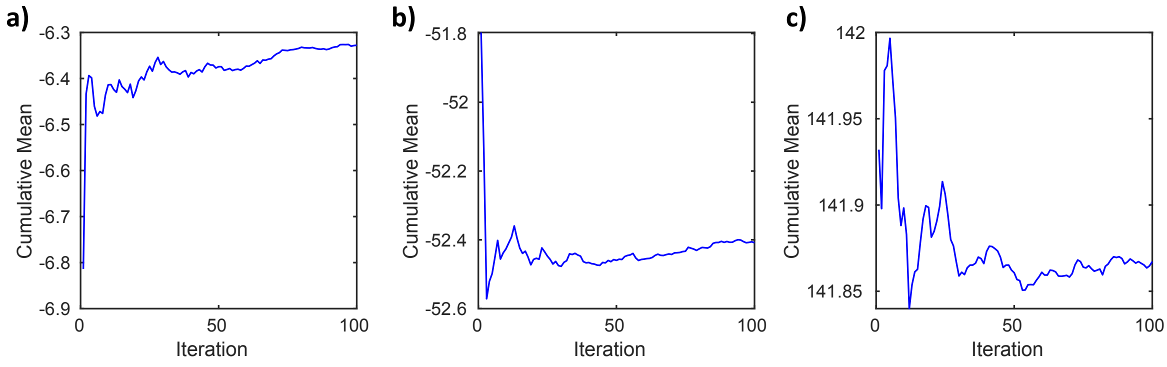

Figure S 8. Cumulative mean convergence plots of $\langle\sigma_{iso}\rangle$ for a) potassium pyruvate C-1, b) potassium pyruvate C-2 and c) potassium pyruvate C-3

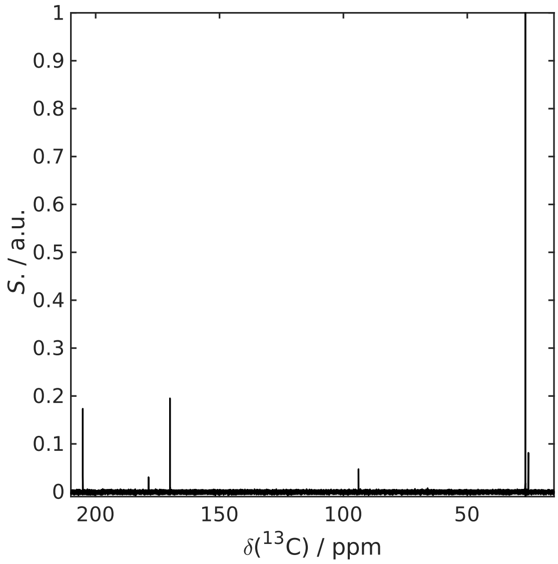

Figure S 9. ^{13}C NMR spectrum of sodium pyruvate in D_2O.

Methodological Considerations and Validation

Several aspects of the proposed methods should be addressed. Most critically, it must be considered that the error in the computed shielding values (Fig. 5b) is relatively large, such that the small changes in chemical shifts between free and bound phosphate species (Fig. 5a) fall within the computational error margin (histogram width in Fig. 4 and the Supporting Information). While the overall pH-dependent trends agree well, the magnitude of the computed error bars limits the ability to reliably interpret small differential values (e.g., Δδ between P_i and P_{PNC} at a given pH). The large uncertainty is due to the large range of ^{31}P chemical shielding values actually averaged (see Fig. S5-S6) as well as the finite number of GIPAW calculations (falling short of experimental averaging). Although, considering this, the correlations presented are within the expected range, a quantitative comparison between the free and bound states cannot be undertaken with sufficient certainty, contrary to pure experimental data (Fig. 2). Although our results clearly demonstrate that the computational approach captures the major spectral features and trends but could benefit from future inclusion of exchange dynamics or mixed-state averaging to refine accuracy. Finally, to ensure the broader applicability of our computational approach and confirm that the agreement observed for CaP PNC was not a singular case or biased by exchange effects, we confirmed its performance on a chemically distinct system, namely sodium pyruvate as extensively displayed in the Supporting Information.

Pyruvate is a well-established benchmark substrate for dDNP and is frequently used as a reference in hyperpolarization studies.[1-3] For further testing of our method, it has two advantages: (i) the chemical shifts of all its resonances across its ^{13}C spectrum are much more dispersed than the ^{31}P chemical shift in phosphate. Thus, the experimental values do not fall within the computational error margin. Besides a second probed nucleus apart from ^{31}P highlights broader applicability. (ii) It can also form Ca^{2+}-dipyruvate clusters in solution as a precursor of solid calcium pyruvate[4] (although not distinctly identified in the literature as PNC). Thus, the Ca-P_i interaction can also be validated.

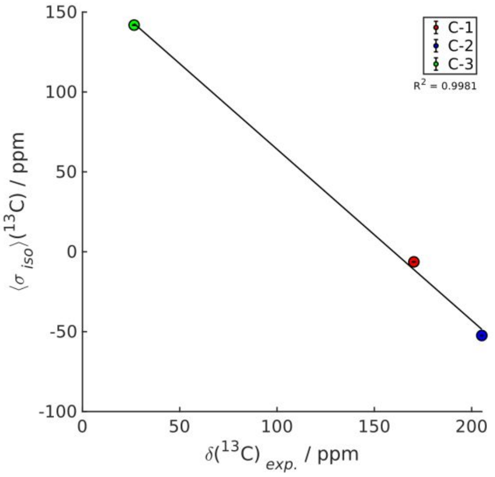

Figure S 10. Correlation plot between measured chemical shift δ and calculated magnetic shielding $\langle \sigma_{iso} \rangle$ of potassium pyruvate C-1 (blue), C-2 (purple) and C-3 (orange). Note that the error bars are within the marker width.

(i) Concerning the first point, using the same methodology applied to CaP PNC, we computed and measured ^{13}C chemical shift for free pyruvate. Due to the presence of several carbon sites, experimental and computational results can also be correlated through a single spectrum. Such an analysis is furthermore important as the pyruvate spectrum is unaffected by possible exchange averaging. The result is shown in Fig. S10, *i.e.*, time-averaged $\langle \sigma_{iso} \rangle$ compared to the experimental chemical shift of the three pyruvate ^{13}C signals. *A* qualitative agreement ($R^2 > 0.99$) was found, which clearly reinforces the robustness of the proposed workflow. All underlying computational and experimental pyruvate data can be found in Supporting Information (Figs. S7-S9).

(ii) Concerning the Ca^{2+} interaction study, we performed dDNP experiments on pyruvate-$^{13}C_1$ injected into a Ca^{2+}-containing buffer. We observed a splitting of the pyruvate line corresponding to the free species and the Ca^{2+}-bound cluster $CaPyr_2$ immediately at the onset of detection. The chemical shift difference was $\Delta\delta$ of -0.38±0.01 ppm. Importantly, the appearance of two distinct lines indicates a system in slow exchange, such that the resonances are not affected by exchange averaging, contrary to the CaP case.[5] All data can be found in the Supporting Information Fig. S11. Repeating the computational workflow for the $CaPyr_2$ cluster, a difference in chemical shielding of $\Delta\langle\sigma_{iso}\rangle$ = 0.39±0.37 ppm was found for the $^{13}C_1$ carboxyl nucleus between free and Ca^{2+}-bound pyruvate (Fig.

S10g-h). Hence, in this case, the differential computational and experimental values matched, even despite significant computational errors.

Together, these two results provide strong validation for the computational approach, demonstrating that it reproduces chemical shifts as well as their perturbations upon calcium coordination correctly, even beyond the specific case of CaP PNC and in the absence of chemical exchange. The ability to achieve similarly high accuracy for a well-characterized system that serves as a key benchmark in dDNP strongly supports our approach to ensemble averaging and solution-state chemical shifts.

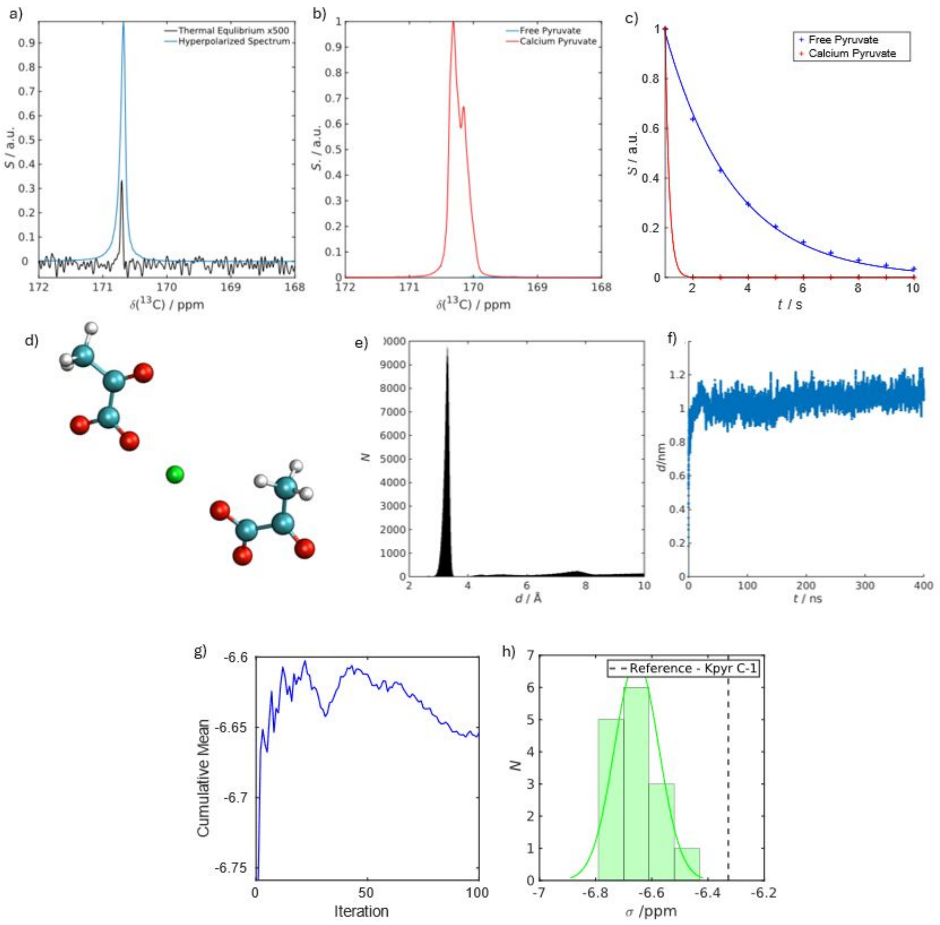

Figure S 11. *Results on Ca^{2+}-pyruvate complexation. a) dDNP results for free pyruvate, the signal enhancement was ca. 15,000. b) The hyperpolarized pyruvate spectrum in the presence of Ca^{2+}, the chemical shift difference between free and bound species (here in slow exchange) is 0.38 ppm. c) Hyperpolarization decay curves for the experiments in panels a and b. d) Simulated, representative low-energy structure of the Ca^{2+}(pyruvate)$_2$ complex. e) The corresponding distance histogram. f) The corresponding RMSD, complex formation takes place after ca. 50 ns. g) The GIPAW bootstrapping iterations. h) $\langle\sigma_{iso}\rangle$ histogram for Ca^{2+}(pyruvate)$_2$ the reference value is derived from Fig. S8.*

References

1. Harris, T.; Eliyahu, G.; Frydman, L.; Degani, H., Kinetics of hyperpolarized 13C1-pyruvate transport and metabolism in living human breast cancer cells. *Proceedings of the National Academy of Sciences* **2009,** *106* (43), 18131-18136.

2. Nelson, S. J.; Kurhanewicz, J.; Vigneron, D. B.; Larson, P. E.; Harzstark, A. L.; Ferrone, M.; Van Criekinge, M.; Chang, J. W.; Bok, R.; Park, I., Metabolic imaging of patients

with prostate cancer using hyperpolarized [1-13C] pyruvate. *Science translational medicine* **2013,** *5* (198), 198ra108-198ra108.
3. Negroni, M.; Guarin, D.; Che, K.; Epasto, L. M.; Turhan, E.; Selimovic, A.; Kozak, F.; Cousin, S.; Abergel, D.; Bodenhausen, G.; Kurzbach, D., Inversion of Hyperpolarized (13)C NMR Signals through Cross-Correlated Cross-Relaxation in Dissolution DNP Experiments. *J Phys Chem B* **2022,** *126* (24), 4599-4610.
4. Paradies, H.; Quitschau, P.; Pischel, I., Structure and properties of calcium pyruvate in aqueous solutions. *Zeitschrift für Physikalische Chemie* **2000,** *214*, 301-311.
5. Turhan, E.; Minaei, M.; Narwal, P.; Meier, B.; Kouril, K.; Kurzbach, D., Short-lived calcium carbonate precursors observed in situ via Bullet-dynamic nuclear polarization. *Commun Chem* **2024,** *7* (1), 210.
